# Characterizing the Immune Landscape of a Syngeneic Mouse Model with Regional Lymph Node Metastasis and Distant Lung Metastasis

**DOI:** 10.3390/cancers18172796

**Published:** 2026-08-28

**Authors:** Deyi Xiao, Wenxuan Zhang, Austin McMasters, Chuanlin Ding, Hong Li, Yi Zou, Alisa Sweazy, Quinn Piamonte, Jun Yan, Kelly McMasters, Haixun Guo, Hongying Hao

**Affiliations:** 1 The Hiram C. Polk, Jr., MD, Department of Surgery, Division of Immunotherapy, School of Medicine, University of Louisville, Louisville, KY 40202, USA; deyi.xiao@louisville.edu (D.X.); chuanlin.ding@louisville.edu (C.D.); alisa.sweazy@louisville.edu (A.S.); jun.yan@louisville.edu (J.Y.); mcmasters@louisville.edu (K.M.); 2 Department of Radiology and Center for Predictive Medicine, School of Medicine, University of Louisville, Louisville, KY 40202, USA; wenxuan.zhang@louisville.edu; 3 Center for Predictive Medicine, School of Medicine, University of Louisville, Louisville, KY 40202, USA; 4 Department of Microbiology and Immunology, School of Medicine, University of Louisville, Louisville, KY 40202, USA; austin.mcmasters@louisville.edu; 5 UofL Health Brown Cancer Center, School of Medicine, University of Louisville, Louisville, KY 40202, USA; hong.li@louisville.edu (H.L.); yi.zou@louisville.edu (Y.Z.); 6 School of Medicine, University of Louisville, Louisville, KY 40202, USA; quinn.losefsky@louisville.edu

**Keywords:** melanoma, lymphatic metastasis, tumor-draining lymph node (TDLN), immune microenvironment, pre-metastatic niche, syngeneic mouse model, B16F10

## Abstract

**Background/Objectives**: Tumor-draining lymph nodes (TDLNs) play a critical role in shaping a pre-metastatic and immunosuppressive niche that facilitates tumor dissemination. However, a robust in vivo model that recapitulates regional lymph node and distant metastasis remains elusive. We sought to establish and characterize an animal model that reliably mimics spontaneous human melanoma progression from primary tumor to regional lymph node and distant organ metastasis. **Methods**: B16F10-Luc2 melanoma cells were subcutaneously implanted from the hock area into the lower medial thigh of C57BL/6J mice to establish a metastatic melanoma mouse model with reliable regional lymph node metastasis and distant metastasis. Tumor growth was monitored over time. ^18^F-fluorodeoxyglucose (FDG) positron emission tomography/computed tomography (PET/CT) and bioluminescence imaging (BLI) were used to track lymph node and lung metastases in vivo. Immunocytochemistry (IHC) was performed to confirm the metastatic melanoma cells in tissues. The immune landscape of metastatic lymph nodes was characterized by Cytometry by Time-Of-Flight (CyTOF). **Results**: This implantation strategy resulted in consistent primary tumor growth accompanied by reproducible metastasis to ipsilateral inguinal lymph nodes (IILN) and subsequent lung metastasis. Multi-modal imaging (PET/CT and BLI) enabled non-invasive monitoring of metastatic progression in vivo. Histological analyses confirmed melanoma cell infiltration in lymph nodes and lung tissues. Notably, CyTOF profiling revealed a distinct immunological remodeling within the TDLN, characterized by immunosuppressive phenotypes in the tumor-draining ipsilateral inguinal lymph node (IILN) compared with naïve nodes, indicative of a potential immunotolerant environment in tumor-bearing mice associated with metastatic progression. **Conclusions**: We present a reproducible and clinically relevant syngeneic melanoma mouse model that recapitulates lymphatic dissemination to regional lymph nodes and distant lung metastasis, and mimics the immunosuppressive phenotypes in the tumor-draining lymph node microenvironment seen in human melanoma patients. This model provides a valuable platform for investigating the mechanisms of lymphatic metastasis and the immunological dynamics of TDLNs, with potential applications in evaluating therapeutic strategies targeting metastatic progression.

## Simple Summary

B16F10-Luc2 melanoma cells were subcutaneously implanted from the hock area into the lower medial thigh of C57BL/6J mice to establish a reproducible and clinically relevant syngeneic melanoma mouse model that recapitulates lymphatic dissemination to regional lymph nodes and distant lung metastasis. ^18^F-fluorodeoxyglucose (FDG) positron emission tomography/computed tomography (PET/CT) enabled non-invasive monitoring of metastatic progression in vivo. Immunocytochemistry (IHC) confirmed the metastatic melanoma cells in tumor-draining lymph nodes (TDLNs) and lung tissue. The immunosuppressive phenotype of the immune cells in the tumor-draining lymph node observed in this model mimics those seen in human melanoma patients. This model provides a valuable platform for investigating the mechanisms of lymphatic metastasis, with potential applications in evaluating therapeutic strategies.

## 1. Introduction

Melanoma is an aggressive form of skin cancer characterized by its highly metastatic potential and rapid progression [1]. Tumor-draining lymph nodes (TDLNs) not only function as pre-metastatic niches to remodel the tumor-induced immunosuppressive microenvironment and facilitate tumor cell metastasis, but also act as key regulators of anti-tumor immunity [2,3]. Reliable in vivo models that faithfully recapitulate the sequential steps of melanoma metastasis, including local invasion, lymphatic spread, and distant colonization, are essential for elucidating the mechanisms of melanoma metastasis and evaluating potential therapeutic interventions targeting metastatic disease. Characterizing the immune landscape of the metastatic lymph node is critical for exploring the responses to immunotherapy.

The most often used melanoma metastasis model is intravenous injection of B16F10 cells in C57BL/6 mice [4]. This model creates highly aggressive, reproducible lung tumor nodules used for studying hematogenous metastasis and testing therapeutic agents [4]. However, this model bypasses early steps of tumor progression and does not adequately model lymphatic spread or the formation of tumor-draining lymph node niches. While subcutaneous and orthotopic implantation models allow for primary tumor development, they frequently exhibit inconsistent or low rates of spontaneous lymph node and distant metastasis, limiting their utility for studying the dynamics of metastatic progression (5, 6). Furthermore, many current models lack the ability to longitudinally monitor metastasis in vivo or to comprehensively characterize the evolving immune microenvironment within metastatic sites [5,6,7,8]. These limitations highlight the need for a robust and reproducible in vivo system that faithfully mimics lymphatic dissemination and enables integrated analysis of metastatic progression and immune remodeling.

To address these limitations, we developed a syngeneic melanoma model designed to more faithfully recapitulate lymphatic dissemination and subsequent distant metastasis. By subcutaneously implanting B16F10-Luc2 cells from the hock region into the lower medial thigh, a site with defined lymphatic drainage to the inguinal lymph nodes, we created a consistent mouse model that enables melanoma cells to metastasize to a tumor-draining lymph node (TDLN), followed by distant metastasis to the lungs. The incorporation of bioluminescence imaging (BLI) and positron emission tomography/computed tomography (PET/CT) further allows for non-invasive, longitudinal monitoring of metastatic progression in vivo. Immunohistochemistry (IHC) was used to confirm the metastatic cells in lymph nodes and the lungs. Importantly, CyTOF profiling revealed a distinct immunological remodeling within metastatic lymph nodes, characterized by altered immune cell composition compared with naïve lymph nodes, indicative of a localized immunosuppressive microenvironment, as seen in human melanoma patients [9]. Collectively, our study offers a robust and clinically relevant platform for investigating the mechanisms of lymphatic metastasis and evaluating therapeutic interventions targeting metastatic disease. It also provides insight into tumor-induced immunological remodeling by comprehensive characterization of the immune microenvironment within metastatic lymph nodes.

## 2. Materials and Methods

### 2.1. Mouse Melanoma Cell Line and Culture

Mouse melanoma cell line B16F10-Luc2 cells (CRL-6475-LUC2) were purchased from American Type Culture Collection (ATCC, Manassas, VA, USA) and maintained in Dulbecco’s Modified Eagle Medium (DMEM) (Corning, Corning, NY, USA) containing 1% penicillin/streptomycin, 10% (*v*/*v*) fetal bovine serum (FBS), and 10 ug/mL Blasticidin (Sigma Aldrich, St. Louis, MO, USA) at 37 °C and 5% CO_2_. Cell passages were kept below 20. The cell line was authenticated by the ATCC cell bank using short tandem repeat (STR) profiling. This cell line was also tested pathogen-free before injection into mice. ^18^F-fluorodeoxyglucose (FDG) was obtained from Siemens PETNET on-site at the University of Louisville.

### 2.2. Melanoma Mouse Model

**Figure 1:**
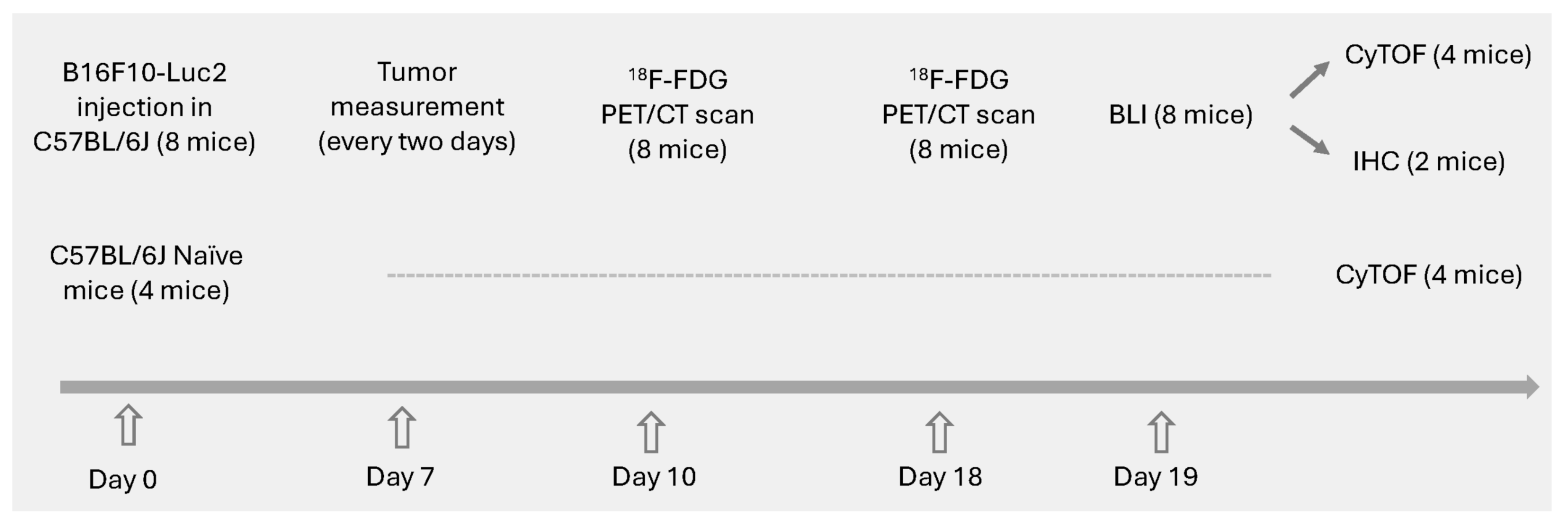
Experiment groups and timeline.

C57BL/6J mice (strain #: 000664) were purchased from Jackson Laboratory (Bar Harbor, ME, USA). Mice that were 7 weeks old were used in this study. The 1 × 10^5^ B16F10-Luc2 cells in 30 µL of phosphate-buffered saline (PBS) were injected as described below (counted as Day 0). Naïve mice were injected with PBS at the same time. Tumors were measured every two days with a caliper after 7 days of B16F10-Luc2 cell implantation. The formula (width × width × length)/2 was used to calculate the tumor volumes (V). Tumors usually reach the endpoint of 15 mm in either width or length within 20 days after tumor cell implantation. The duration of the study was about 20 days. The experiment grouping and timeline are shown in Figure 1. All the animal procedures were approved by the University Committee for Animal Welfare. All animals were maintained under specific pathogen-free conditions and handled in accordance with the protocol approved by the University Committee for Animal Welfare (UCAW) (protocol number: 25515).

### 2.3. Procedure of Subcutaneous Implantation from the Hock Area into the Lower Medial Thigh of Mouse

**Figure 2:**
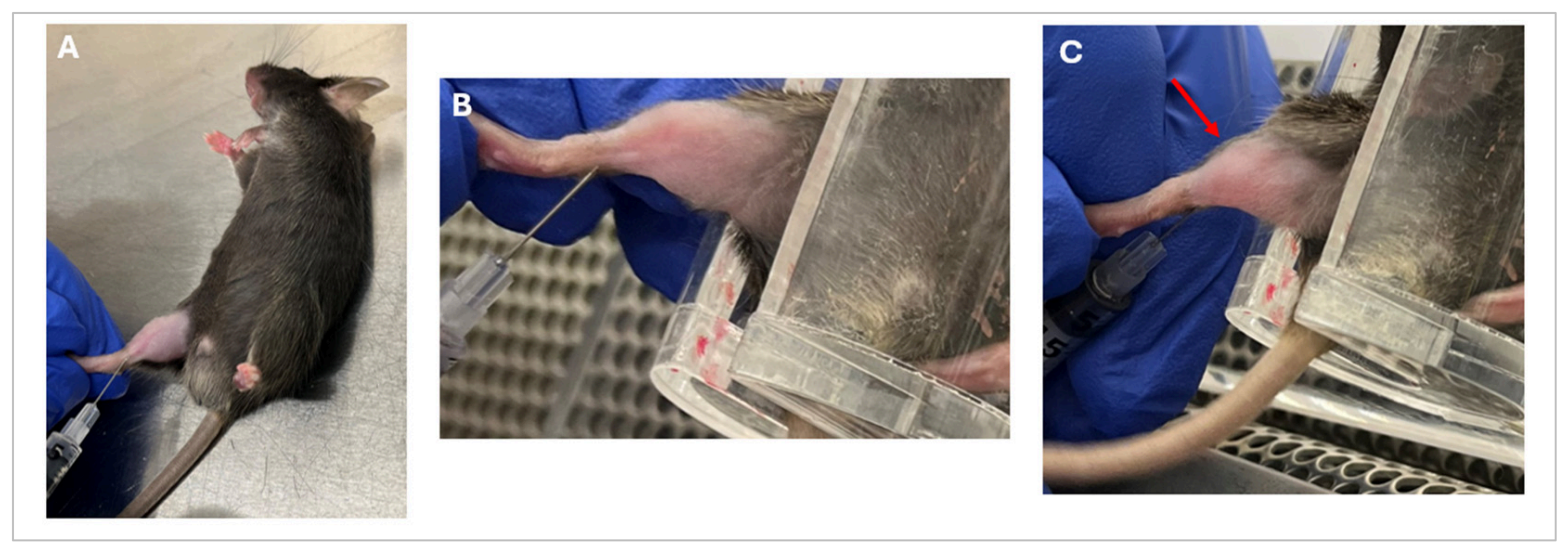
B16F10-Luc2 tumor cell injection site. (**A**) Needle starting point. (**B**) Enlarged picture of (**A**). (**C**) Subcutaneous bump (red arrow) after tumor cell injection.

Mice were shaved in the hock area of the left hind foot (from supine position) after being anesthetized with 1.5% isoflurane (Figure 2A). After shaving, the mice were placed on a restrainer with the left foot exposed outside (Figure 2B). A 1 mL insulin syringe with a 30-gauge needle was used for injection. The needle was inserted just above the ankle and directed proximally. With one hand holding the foot, the other hand was used to hold the syringe and insert the needle with the beveled edge facing to the left and upward. The needle was inserted into the subcutaneous tissue of the lower medial thigh before injecting slowly. The needle was then withdrawn slowly to avoid leakage of injected cells. A subcutaneous bump (in Figure 2C) can be seen after tumor cell injection. 

### 2.4. ^18^F-FDG PET/CT Scan

^18^F-FDG (3.7 MBq in 0.2 mL PBS) was intraperitoneally (IP) injected into each mouse, and a 10 min PET scan (Bruker Molecubes β-CUBE, Ghent, Belgium) was acquired at 45 min post-dose injection, followed by a 5 min high-resolution CT scan (Bruker Molecubes X-CUBE, Ghent, Belgium). PET/CT data was analyzed with PMOD (v4.405).

### 2.5. Bioluminescence Imaging (BLI)

BLI was performed the next day after the ^18^F FDG PET/CT scan. Spectral Instrument Imaging (Bruker, Tucson, AZ, USA) was used for BLI. Mice were humanely sacrificed. Tissues (lymph nodes, lung, liver, and spleen) were harvested for BLI before further analysis.

### 2.6. Mouse Tissue Processing

For CyTOF analysis, collected inguinal lymph nodes were placed in DMEM (Corning life sciences, Corning, NY, USA) containing 10% FBS. Lymph node samples were mechanically disrupted into small pieces using 1 mL pipette tips. Further mechanical shattering was done using syringe columns. The single-cell suspensions were then filtered through a 70 μm cell strainer, washed, lysed in Ammonium–Chloride–Potassium (ACK) buffer, re-filtered through a 70 μm cell strainer, and cryopreserved in 90% FBS and 10% DMSO. For immunohistochemistry staining, collected lymph nodes were placed in 10% formalin until further embedding.

### 2.7. Immunohistochemistry (IHC) Staining

Formalin-fixed paraffin-embedded tissues were cut into 4 µm-thick sections. Standardized IHC procedures were applied to stain the metastatic melanoma cells in lymph node and lung tissue by VitroVivo Biotech (Rockville, MD, USA). The primary anti-Melan A antibody (ab210546) was from Abcam Inc. (Waltham, MA, USA). Images were captured using a Panoramic Desk Slide Scanner (3dhistech, Budapest, Hungary).

### 2.8. Mass Cytometry (CyTOF) Data Acquisition and Data Analysis

CyTOF was performed as described before [10]. Briefly, after the single-cell suspensions were thawed, cell permeabilization and staining were performed following the protocol from Fluidigm (Boston, MA, USA). Before acquisition, cells were suspended in a 1:9 cell acquisition solution (Fluidigm catalog# 201241): EQ 4 element calibration beads (Fluidigm catalog #201078) at an appropriate concentration, with no more than 500 events per second. Data acquisition was completed on the CyTOF Helios system (Fluidigm, Boston, MA, USA). Flow Cytometry Standard (FCS) files were normalized with the Helios instrument work platform (FCS Processing) based on the calibrated bead signal. FlowJo software (Version 10.10.1) was used for CyTOF data analysis. Total CD45+ cells were gated after beads, doublets, and dead cells were removed. FlowJo Plugins platform was used to perform t-distributed stochastic neighbor embedding (tSNE) and FlowSOM clustering for CyTOF data. tSNE analysis was performed on a concatenated fcs file created from all samples, and each sample contributed an equal number of CD45+ cells. Different immune cell populations were defined by the expression of specific surface markers. Antibodies used for CyTOF are listed in Supplemental Table S1.

### 2.9. Statistical Analysis

Student’s *t*-test was used to calculate the significance between the groups. Graphical representations were generated using GraphPad Prism version 11.0 (GraphPad Software, San Diego, CA, USA). All graph bars are expressed as mean ± standard deviation (SD). Significance was assumed to be reached at *p* < 0.05. The *p*-value was presented as follows: * *p* < 0.05, ** *p* < 0.01, and *** *p* < 0.001.

## 3. Results

### 3.1. Primary Tumor Growth After Subcutaneous Implantation of B16F10-Luc2 Cells from the Hock Area into the Lower Medial Thigh of C57BL/6J Mice

**Figure 3:**
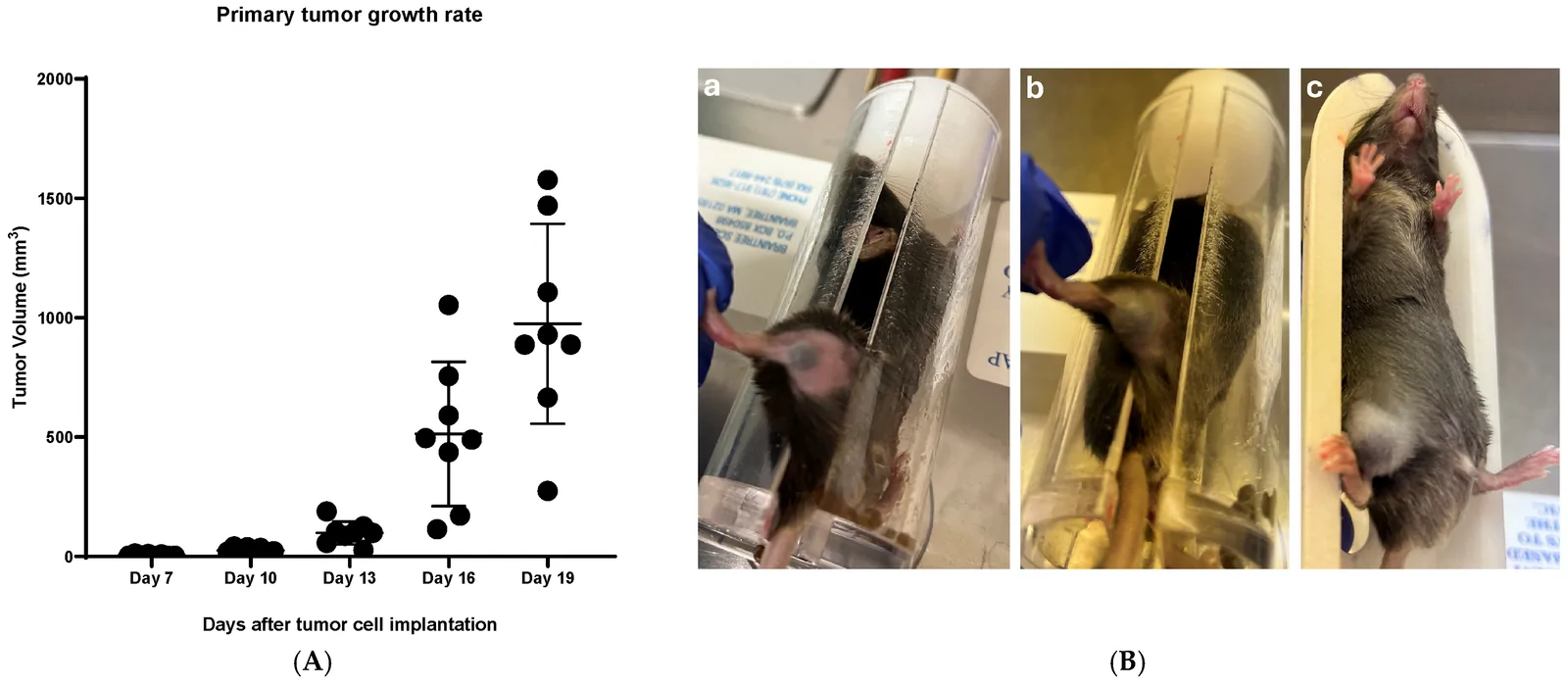
(**A**) Primary tumor growth rate (n = 8, bars represent mean ± SD). (**B**) Representative pictures of tumor size after day 10 (a), 13 (b), and 16 (c) of B16F10-Luc2 tumor cell implantation.

The B16F10 melanoma model is the most widely used syngeneic melanoma model. Our research interests focus on characterizing the immune features of the lymph node in this mouse model and exploring how the immune status in the lymph node affects distant metastasis. To establish a melanoma model capable of recapitulating lymphatic dissemination, B16F10-Luc2 cells were subcutaneously implanted from the hock region into the lower medial thigh of C57BL/6J mice, a site with defined lymphatic drainage to the inguinal lymph nodes. Around 7 days after B16F10-Luc2 cell implantation, a primary tumor began to appear. Tumor volume was recorded every two days starting on day 7 after tumor cell implantation (Figure 3A). Tumor growth was slow during the first 13 days after tumor cell implantation, but usually grew to approximately 15 mm (width) × 15 mm (length) within the next 7 days (Figure 3B). Although there was some variation in growth rate of the primary tumors among the mice, all the primary tumors progressed over time, demonstrating the reproducibility of primary tumor establishment by subcutaneous implantation of B16F10-Luc2 cells from the hock area into the lower medial thigh of C57BL/6J mice (Figure 3A,B). During the study period, no changes in animal behaviors, such as aggressiveness, reduced grooming, isolation, and altered food and water intake, were observed.

### 3.2. Non-Invasive ^18^F-FDG PET/CT Scanning Enables Longitudinal Tracking of Metastatic Melanoma Cells in the Regional Inguinal Lymph Node and Lung

**Figure 4:**
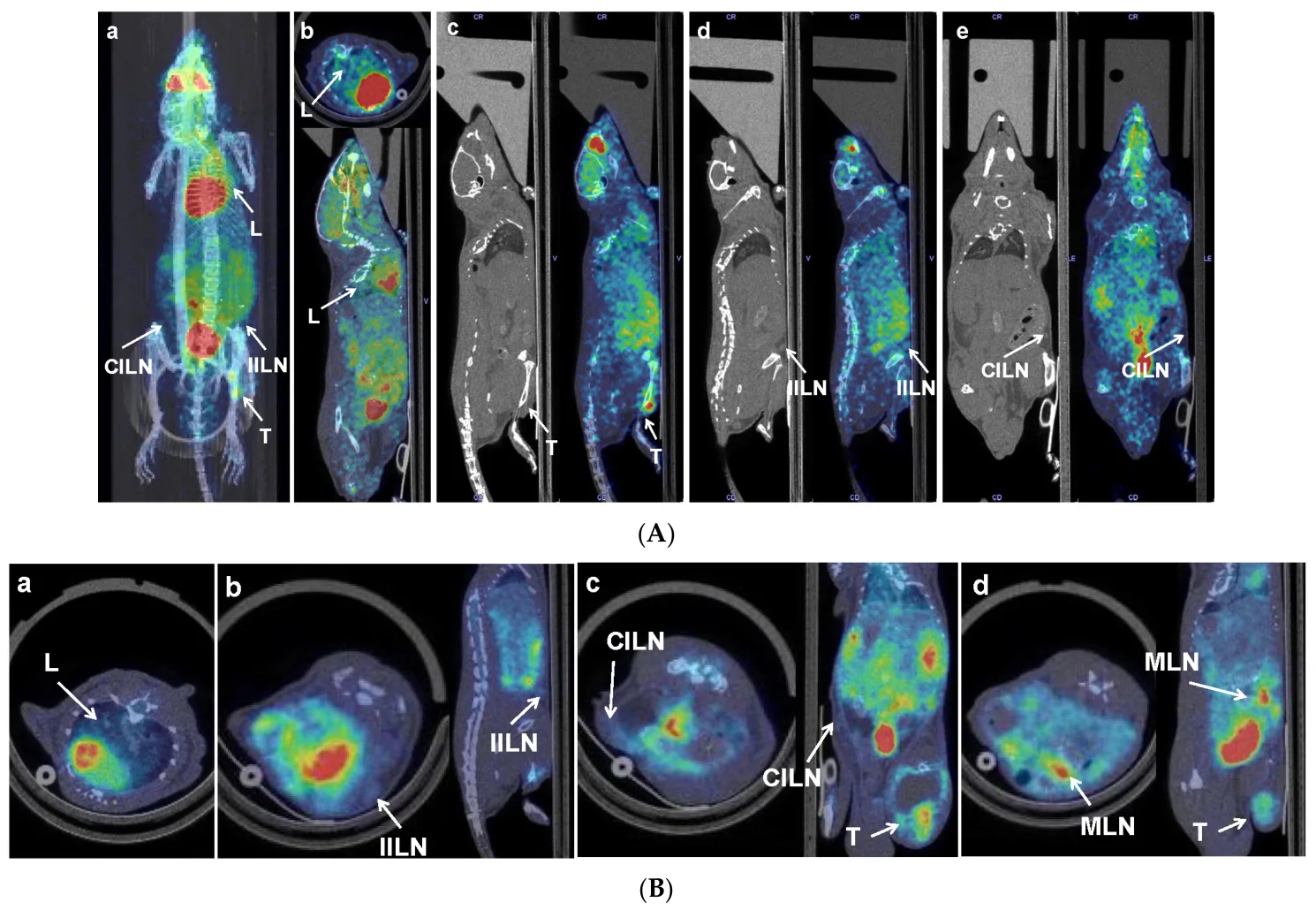
(**A**) ^18^F-FDG PET/CT imaging revealed upregulated signals in tumor (T), lungs (L), and ipsilateral inguinal lymph node (IILN), but not the contralateral inguinal lymph node (CILN) at 10 days post tumor cell implantation. (a): whole body; (b): transverse and sagittal; (c): Sagittal CT and PET/CT; (d): Sagittal CT and PET/CT; (e): Coronal CT and PET/CT. (**B**) 18F-FDG PET/CT imaging revealed upregulated signals in tumors (T), lungs (L), ipsilateral inguinal lymph node (IILN), contralateral inguinal lymph node (CILN), and mesenteric lymph node (MLN) at 18 days post tumor cell implantation. (a): transverse; (b): transverse and sagittal; (c): transverse and coronal; (d): transverse and sagittal.

We took advantage of the high sensitivity of ^18^F-FDG PET/CT for detecting metastatic spread of tumor cells. On day 10 of B16F10-Luc2 cell implantation, most of the primary tumors were around 3–4 mm (width) × 3–4 mm (length). Mice were subjected to ^18^F-FDG PET/CT scanning under anesthesia. Ipsilateral inguinal lymph node (IILN) and lung metastasis were detected by ^18^F-FDG PET/CT scanning (Figure 4A). Contralateral inguinal lymph nodes (CILN) did not show ^18^F-FDG uptake (Figure 4A). There were no metastatic tumor cells detected in other tissues (liver and spleen) on day 10 after B16F10-Luc2 cell implantation by ^18^F-FDG PET/CT scanning. On day 18 after B16F10-Luc2 cell implantation, most of the primary tumors reached around 11–14 mm (width) × 11–14 mm (length). ^18^F-FDG PET/CT was performed again to longitudinally monitor metastasis. The uptake of ^18^F-FDG was observed in the lungs and IILNs in all eight mice. Representative ^18^F-FDG uptake in the lung and IILNs is shown in Figure 4B. Positive ^18^F-FDG PET signals in CILNs were only found in two of the mice (Figure 4B). One mouse demonstrated ^18^F-FDG uptake in the lung, IILN, and mesenteric lymph node (MLN) (Figure 4B), but not in the CILN. There were no metastases detected in the liver or spleen on day 18 of tumor cell implantation by ^18^F-FDG PET/CT scanning. For the positive ^18^F-FDG signals in IILN and CILN, post-imaging ex vivo BLI and IHC staining confirmed the metastasis of melanoma cells. Detection of ^18^F-FDG uptake in IILN and lung of all eight mice by PET/CT showed consistent melanoma metastasis to the draining inguinal lymph nodes and lung in this model. Metastasis to other lymph nodes, such as CILN and mesenteric LN, was occasionally seen in this model.

### 3.3. Non-Invasive BLI Confirmed the Presence of Metastatic Tumor Cells in Regional Inguinal Lymph Node and Lung

**Figure 5:**
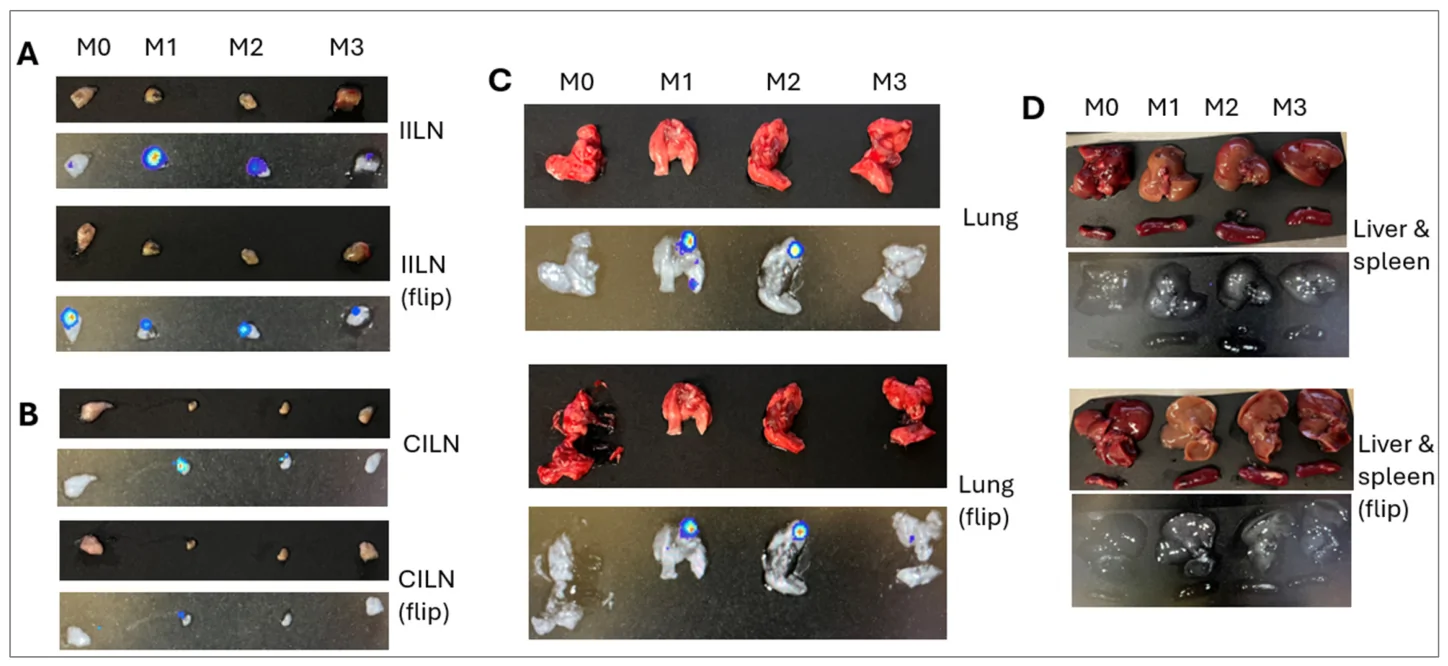
Bioluminescent imaging (BLI) showed metastatic tumor cells in all IILNs (**A**), two CILNs (**B**), and three lungs (**C**). No metastatic tumor cells were detected in liver and spleen by BLI (**D**).

Because ^18^F-FDG PET/CT can be false-positive due to glucose uptake or tissue inflammation, we used BLI, another non-invasive imaging modality that can specifically track luciferase-labeled tumor cells without radiotracers. Our initial data showed that 1.25 × 10^5^ of B16F10-Luc2 cells can be detected by BLI in vitro in tissue culture plates. However, neither IILN nor lung metastasis can be detected by BLI in vivo after 10 days or 19 days of subcutaneous implantation of B16F10-Luc2 cells from the hock area into the lower medial thigh in C57BL/6J mice. A possible explanation is that the luciferase-labeled cells were located too deeply in the tissues to allow detection. Therefore, on day 19 after B16F10-Luc2 cell implantation, one day after ^18^F-FDG PET/CT scanning, when radiotracers had decayed, we performed BLI after the mice were euthanized. Lung, liver, spleen, and the inguinal lymph nodes from the ipsilateral and contralateral sides of the primary tumor were all collected. BLI was performed in vitro on harvested tissues within one hour of euthanization before luciferin clearance. In order to avoid the luminescent signals being covered by the thick tissues, BLI was performed on one side and on the flip side. The results showed that all the IILNs from the mice implanted with B16F10-Luc2 cells were positive by BLI (Figure 5A), confirming all the positive IILNs detected by ^18^F-FDG PET/CT. Consistent with the results of ^18^F-FDG PET/CT, BLI showed luciferase signals in some, but not all, of the CILNs in mice (Figure 5B). For lung tissues from the mice implanted with B16F10-Luc2 cells, even though all mice showed upregulated signals in the lung by ^18^F-FDG PET/CT, there was one mouse (M0 in Figure 5C) without a lung luciferase signal by BLI. All the other mice showed lung metastasis by BLI (Mice M1, M2, and M3 in Figure 5C—lung flip). Consistent with the results from ^18^F-FDG PET/CT scanning, BLI also did not detect any luciferase-labeled tumor cells in the liver or spleen (Figure 5D). Once again, these results confirmed the metastatic tumor cells in the lymph nodes and lungs by ^18^F-FDG PET/CT scanning.

### 3.4. Metastatic Tumor Cells in IILN and Lung Were Confirmed by IHC

**Figure 6:**
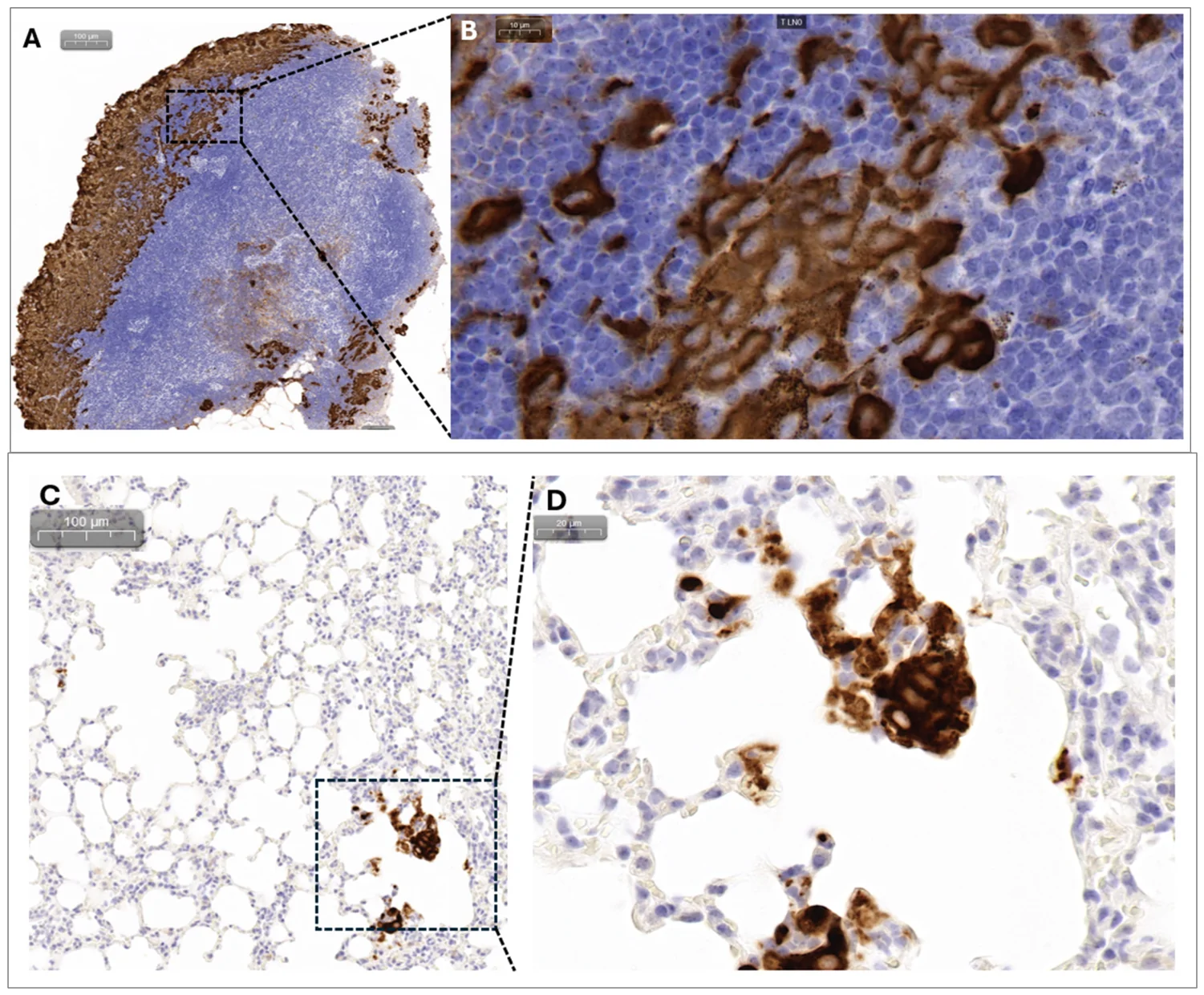
Metastatic melanoma cells confirmed by immunohistochemistry (IHC) using anti-Melan A antibody in mouse inguinal lymph node ((**A**): 8X and (**B**): 80X) and in lung ((**C**): 10X, (**D**): 40X).

After the mice were euthanized, we randomly selected two from the eight mice and collected inguinal lymph node and lung tissues to perform IHC. Metastatic melanoma cells in the inguinal lymph nodes were confirmed by IHC staining with anti-Melan A antibody (Figure 6A,B). Similarly, lung metastases were also confirmed by IHC (Figure 6C,D).

### 3.5. CyTOF Profiling Revealed a Distinct Immunological Remodeling Within Metastatic Lymph Nodes Characterized by Immunosuppressive Microenvironment in IILNs and Immune Plasticity in CILNs in Tumor-Bearing Mice

**Figure 7:**
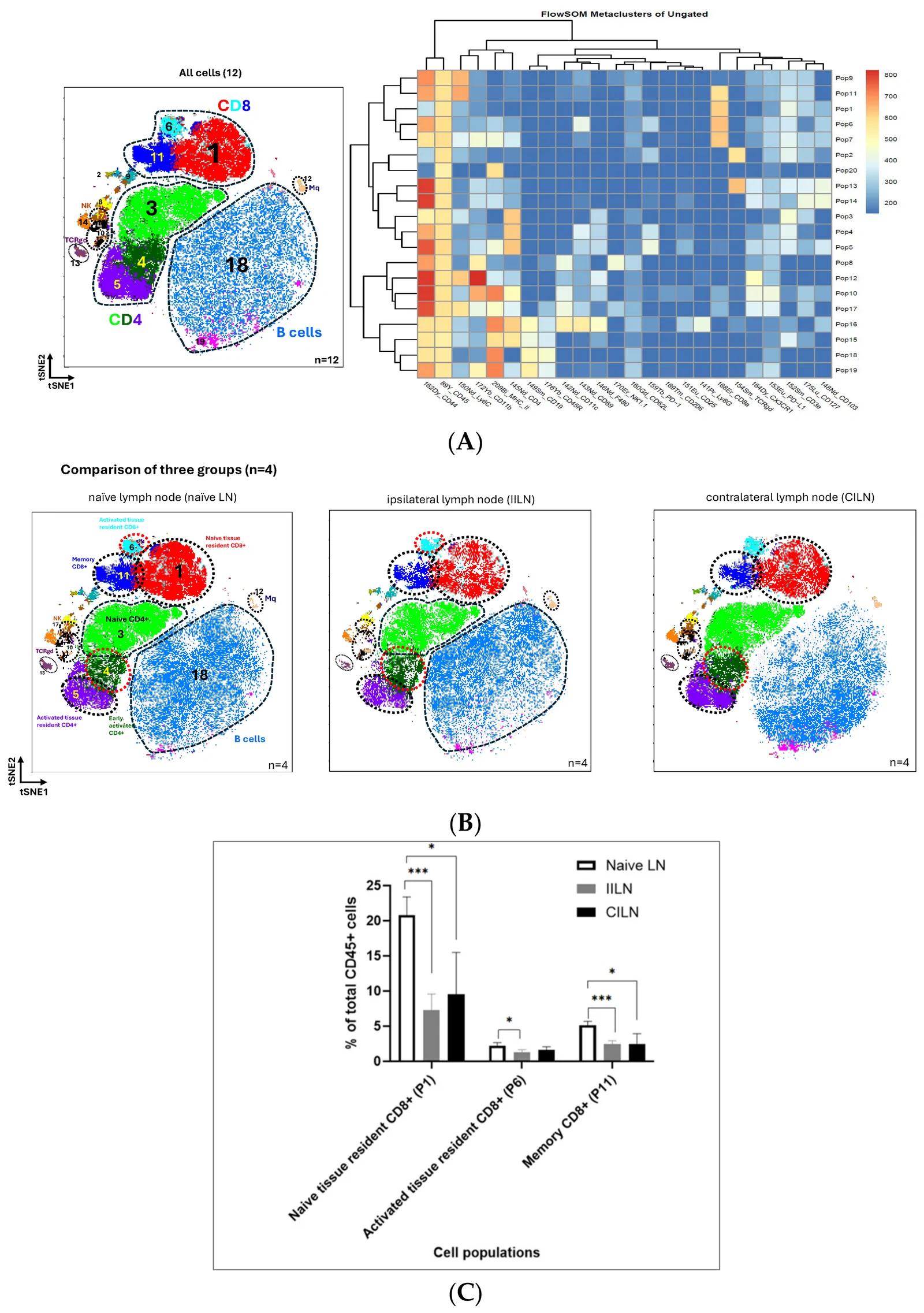

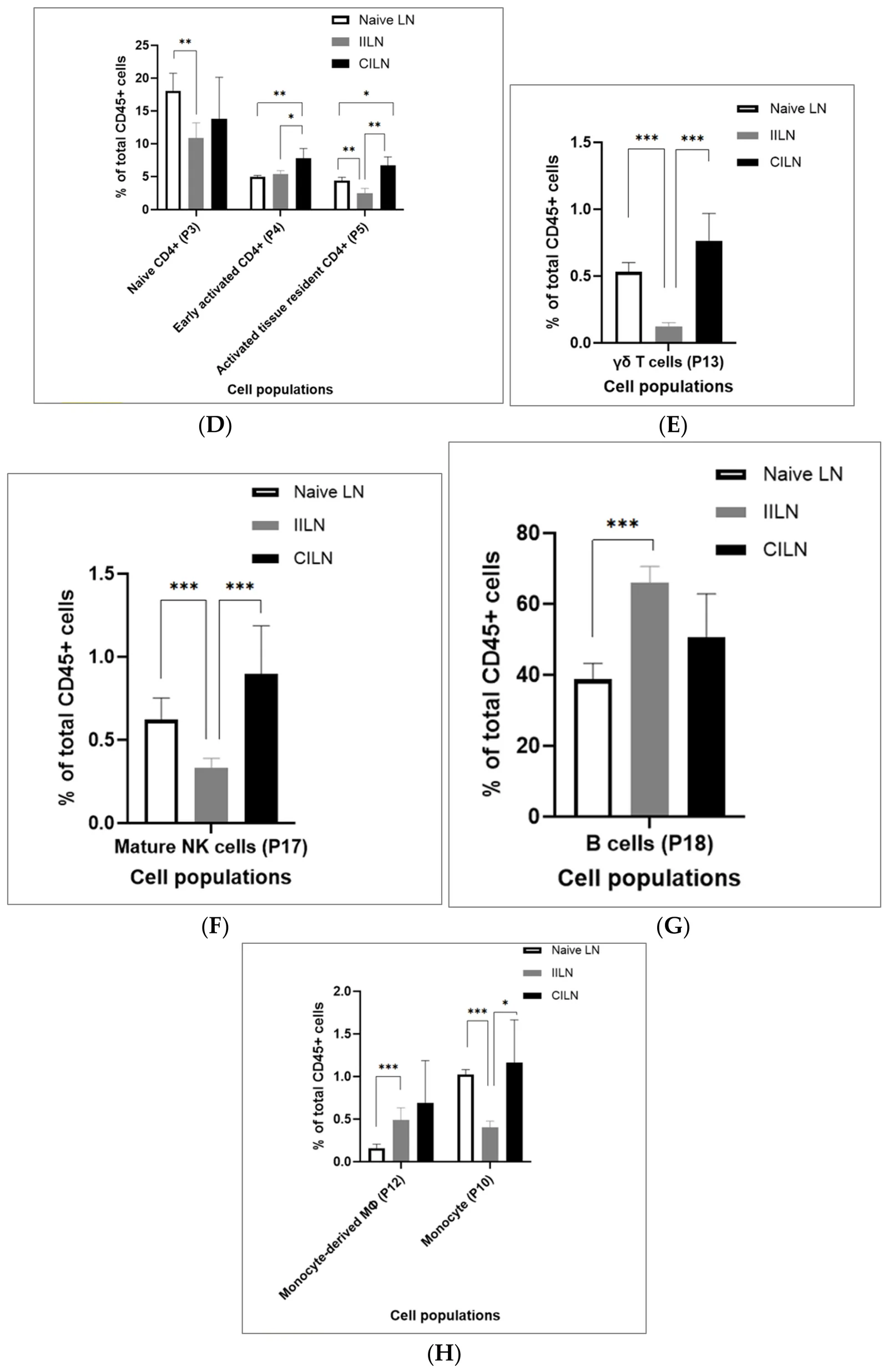
(**A**) viSNE analysis of CyTOF immunophenotyping of lymph node cells from naïve mice, IILN and CILN cells from B16F10-Luc2 cell-implanted mice. All samples combined (n = 12, left. Numbers 1-20 represent different cell populations.). FlowSOM clustering into 20 immune cell subsets, shown as a normalized expression heatmap (right). (**B**) Analysis of CyTOF immunophenotyping in lymph node cells from naïve mice and IILN and CILN cells from B16F10-Luc2-implanted mice. (**C**–**H**) CD8+ T cells (**C**), CD4+ T cells (**D**), γδ T cells (**E**), Mature NK cells (**F**), B cells (**G**), macrophages and monocytes (**H**) in naïve lymph node (naïve LN), ipsilateral lymph node (IILN), and contralateral lymph node (CILN) of tumor cell-implanted mice. (* *p* < 0.05, ** *p* < 0.01, *** *p* < 0.001).

The immune microenvironment denotes the immune response to tumor cells. In order to characterize the immune features of the lymph node, single-cell suspensions were collected from IILNs and CILNs in B16F10-Luc2 cell-implanted mice and naïve inguinal lymph nodes from naïve mice. CyTOF was performed on four samples from each of the groups to compare the immune profile from the IILNs and CILNs of B16F10-Luc2 cell-implanted mice with that of the naïve mice. Figure 7A,B showed the viSNE analysis of CyTOF immunophenotyping of IILN and CILN cells from B16F10-Luc2 cell-implanted mice and naïve node cells from naïve mice. Twenty different immune cell subsets were clustered by FlowSOM clustering analysis (Figure 7A,B). Among them, populations 7, 15, 16, and 20 (p7, p15, p16, and p20) had fewer than 500 cells. They are not shown in Figure 7A,B. All three major CD8+ T cell sub-populations, including naïve tissue-resident CD8+ (p1), activated tissue-resident CD8+ (p6), and memory CD8+ (p11), were all significantly lower in IILNs of tumor cell-implanted mice compared with those in naïve nodes from control mice (Figure 7C). In CILNs of tumor cell-implanted mice, naïve tissue-resident CD8+ (p1) and memory CD8+ (p11) were also significantly lower compared with those in naïve nodes (Figure 7C). The reduced CD8+ T cell frequency in IILNs of tumor cell-implanted mice suggested a potentially immunosuppressive environment in TDLNs.

Among the major CD4+ T cell populations, naïve CD4+ T cells (p3) and activated tissue-resident CD4+ T cells (p5) were all significantly reduced in IILN in tumor cell-implanted mice compared with those in naïve inguinal lymph nodes (Figure 7D). However, in CILNs of tumor cell-implanted mice, early activated CD4+ (p4) and activated tissue-resident CD4+ (p5) were all increased compared with those in naïve inguinal lymph nodes (Figure 7D). These results showed the plasticity of CD4+ T cells in CILNs of tumor cell-implanted mice.

γδT cells (p 13) were significantly lower in IILNs of tumor cell-implanted mice compared with those in naïve nodes. However, γδT cell populations in the CILNs of tumor cell-implanted mice were much greater compared with those in the IILNs (Figure 7E).

Similarly, mature natural killer (NK) cells (p17) were significantly reduced in IILNs of tumor cell-implanted mice compared with those in naïve nodes, but this population of NK cells was much higher in the CILNs of tumor cell-implanted mice compared with those in the IILNs (Figure 7F).

B cells (p 18) were higher in IILNs and CILNs of tumor cell-implanted mice compared with those in the naïve nodes. But it was significant only in IILNs of tumor cell-implanted mice compared with those in the naïve nodes (Figure 7G).

IILNs and CILNs of tumor cell-injected mice had more monocyte-derived macrophages (p 12) than those in naïve nodes, but it was more prominent in IILNs of tumor cell-injected mice (Figure 7H). Monocytes (p10) were reduced in IILNs of tumor cell-injected mice compared with naïve nodes, but they were higher in CILNs than in IILNs of tumor cell-injected mice (Figure 7H).

These data suggest that an immunosuppressive microenvironment was established in TDLNs of B16F10-Luc2 cell-implanted mice, evidenced by significantly reduced CD8+ T cells, γδT cells, and NK cells, as well as substantially increased monocyte-derived macrophages. On the other hand, CILNs of tumor cell-implanted mice showed increased early activated CD4+ T cells, tissue-resident CD4+ T cells, and NK cells, indicating the potential immune-responsive capabilities in CILNs.

## 4. Discussion

In this study, we used subcutaneous implantation of B16F10-Luc2 cells from the hock area into the lower medial thigh of C57BL/6J mice and established a syngeneic melanoma mouse model with consistent primary tumor growth, reproducible regional inguinal lymph node metastasis, and distant lung metastasis. Non-invasive, multi-modality imaging techniques (PET/CT and BLI) effectively tracked metastatic progression, while IHC confirmed melanoma cell infiltration in both lymph nodes and lung tissues. More importantly, high-dimensional CyTOF profiling revealed profound immunological remodeling in IILNs, including reduced CD8+ T cells, γδ T cells, and NK cells, with increased monocyte-derived macrophages. Among them, anti-tumor Ly6C+ naïve tissue-resident CD8+ T cells (p1) were significantly reduced in IILNs of tumor cell-implanted mice compared with those in naïve mice (*p* < 0.001, Figure 7C) [11]. These immune profiles imply the development of an immunosuppressive microenvironment in the IILNs of tumor cell-injected mice relative to naïve controls. In human melanoma patients, we also observed significantly reduced CD8+ T cells and NK cells in sentinel lymph nodes with melanoma metastasis [9]. These findings indicate an immunosuppressive microenvironment in TDLN in this model is similar to that seen in human melanoma patients [3,9]. Compared to other B16F10 metastasis models, traditional subcutaneous flank injections often produce inconsistent or limited spontaneous lymphatic metastasis [12,13]. The intravenous (tail-vein) model primarily results in hematogenous lung metastasis, bypassing the early step of regional lymph node metastasis [14,15]. Footpad or ear injections have been used for orthotopic-like lymphatic spread, but they do not establish reliable distant metastasis and can introduce variability due to anatomical constraints [5,7,16,17]. In contrast, our model of tumor cell implantation from the hock area into the subcutaneous tissue of the lower medial thigh uniquely provides reliable lymphatic drainage to the tumor-draining inguinal nodes and distant lung metastasis. The immunosuppressive phenotypes in the TDLNs in this model resemble those seen in human melanoma patients. This model provides a unique tool for pre-clinical animal studies, especially tumor metastasis through the lymphatic route.

Emerging evidence suggests that TDLNs play a pivotal role in shaping systemic immune responses, often promoting immune tolerance that facilitates metastasis [2,18]. CyTOF profiling in our model revealed that the population of tumor-killing CD8+ T cells and NK cells was significantly lower in the tumor-draining inguinal lymph nodes compared to normal nodes. Furthermore, increased monocyte-derived macrophages (pro-tumor macrophages) create a pre-metastatic niche in IILNs [19,20]. Metastatic spread in this model follows a lymphatic route, beginning with tumor cells entering afferent lymphatics from the primary site to the TDLN. Metastasis to the lungs may take place by simultaneous hematogenous spread, although direct lymphatic–venous connections or further lymphatic remodeling could contribute [21,22,23]. The observed immunosuppressive remodeling in TDLNs suggests that systemic factors, such as tumor-derived exosomes, cytokines, or altered immune cell function, may promote immune tolerance beyond the immediate draining site [24,25,26,27,28]. Tumor-induced lymphangiogenesis and increased lymph flow in TDLNs, as described in other B16F10 systems, probably enhance cell transport and boost the pre-metastatic niche [29,30]. This aligns with emerging evidence that lymph node colonization can epigenetically rewire tumor cells to evade NK cells, expand regulatory T cells, and suppress effector immunity, thereby facilitating distant organ tropism to distant sites [31,32].

B cells are the most abundant cell population in LN tissue. CyTOF profiling demonstrated significantly higher B cell populations in our model. This is consistent with most of the other reports that significant B cell expansion and activation are usually seen with tumor progression [10,33,34]. Even though there are very few NK cells and γδT cells in the LN, they have important roles in anti-tumor response. Studies have shown that lymph node NK cells can efficiently lyse tumor cells and inhibit metastasis of B16 melanoma cells to the draining lymph node [35]. In vivo photolabelling of tumor-infiltrating cells in a mouse model showed that a substantial number of γδT cells were egressed from the tumor site to the TDLN. The level of IFN-γ was decreased in those egressed γδT cells. Communication of γδT cells in tumor tissue and in LN may contribute to the immune response of the host [36]. Another interesting finding is that, in CILNs of tumor cell-implanted mice, we observed reduced CD8+ T cells, similar to those in the IILNs of tumor cell-implanted mice, suggesting decreased anti-tumor immunity in CILNs. We also noted increased early activated CD4+ T cells, tissue-resident CD4+ T cells, and NK cells in CILNs of tumor cell-implanted mice, indicating the potential immune-responsive capabilities in CILNs. This provides an opportunity to modulate the immune environment in other lymph nodes to fight against tumor cells.

Whether we can monitor metastatic progression in vivo longitudinally is an important aspect of evaluating the effectiveness of animal models. PET/CT and BLI were used to track metastatic progression in this model. BLI can specifically track luciferase-labeled tumor cells. But it is a 2-D imaging modality. It is limited in imaging deeper tissues and monitoring metastases. Even though ^18^F FDG PET/CT does not specifically detect only tumor cells, it can provide 3D structured imaging and is a powerful tool for translational studies [37]. Due to the tiny structure of the lymph node and metastasis deeper in the lung tissue, BLI can barely detect lymph node and lung metastasis in vivo in our model. However, PET/CT can detect lymph node and lung metastasis as early as 10 days after tumor cell implantation when the primary tumor size is about 3–4 mm (width) × 3–4 mm (length). This imaging technique can conveniently track the metastatic tumor cells in lymph nodes and in other tissues in vivo longitudinally.

Our model offers several benefits for metastasis studies, including reproducible lymphatic drainage to specific regional lymph nodes, enabling detailed investigation of the tumor–TDLN–distant organ immune axis. This approach better mimics spontaneous metastatic pathways observed in human melanoma, where TDLNs are known to play a pivotal role in metastatic progression and immune evasion. It provides a valuable platform for evaluating anti-metastatic therapies targeting lymphatic dissemination and the immunosuppressive microenvironment. Our research group is currently using this model to explore T cell malfunctions in TDLNs and how to reprogram the immunosuppressive microenvironment in TDLNs in response to metabolites and immunotherapeutic reagents. However, there are some limitations of this model. The first limitation is that it relies on the highly invasive nature of the implanted cells, such as the B16F10 cells used here. For a less invasive murine melanoma cell line, such as YUMM1.7, when cells were implanted from the hock into the lower medial thigh, we also observed metastasis in IILNs, but no CILN metastasis or lung metastasis (unpublished data). Second, for other cancer types, such as breast cancer, we are not sure that implantation in the same region will result in regional and distant metastasis. Third, while the model captures key aspects of lymphatic and distant metastasis, it may not fully represent the heterogeneity of metastatic mechanisms. Future longitudinal studies to investigate the temporal evolution of the immune landscape in TDLN and distant sites will deepen our understanding of metastatic mechanisms. Lastly, integrating functional analysis on specific immune cell types with CyTOF data could provide a more comprehensive understanding of the immune landscape.

## 5. Conclusions

We present a reproducible and clinically relevant syngeneic melanoma mouse model that reliably induces regional lymph node and distant lung metastasis. This model recapitulates key aspects of lymphatic dissemination and metastatic progression observed in human melanoma. This model represents a key innovation that facilitates consistent lymphatic dissemination and enables detailed investigation of tumor–TDLN–distant organ interactions. Despite certain limitations, this model offers distinct advantages in studying the immunological and mechanistic underpinnings of metastasis, providing a clinically relevant platform for translational research and immunotherapeutic evaluation.

## Data Availability

The data presented in this study are available in the article.

## Supplementary Materials

The following supporting information can be downloaded at: https://www.mdpi.com/article/10.3390/cancers18172796/s1; Table S1: List of CyTOF used antibodies.

## Abbreviations

The following abbreviations are used in this manuscript:

ACK	Ammonium–chloride–potassium
ATCC	American Type Culture Collection
BLI	Bioluminescence imaging
CILN	Contralateral inguinal lymph nodes
CyTOF	Cytometry by Time-Of-Flight
DMEM	Dulbecco’s Modified Eagle Medium
FBS	Fetal bovine serum
FCS	Flow cytometry standard
FDG	Fluorodeoxyglucose
IHC	Immunohistochemistry
IILN	Ipsilateral inguinal lymph nodes
IP	Intraperitoneally
MLN	Mesenteric lymph node
NK	Natural killer cells
PET/CT	Positron emission tomography/computed tomography
PBS	Phosphate-buffered saline
SD	Standard deviation
STR	Short tandem repeat
TDLN	Tumor-draining lymph node
tSNE	t-distributed stochastic neighbor embedding
