# Supplementary material for: Characterizing the Immune Landscape of a Syngeneic Mouse Model with Regional Lymph Node Metastasis and Distant Lung Metastasis
Source: Cancers (Basel). 2026 Aug 28;18(17):2796. doi: 10.3390/cancers18172796 (PMC13565651; doi:10.3390/cancers18172796)
Supplement: Supplementary file 1 [file cancers-18-02796-s001.zip › cancers-4500729-supplementary.pdf]

Supplementary Table S1. List of CyTOF used antibodies.

	Antibody Name	Source	Cat#
1	Anti-Mouse CD45 (30-F11)-89Y—100 Tests	FDM	Fluidigm# 3089005B
2	Anti-Mouse Ly-6G (1A8)-141Pr—100 Tests	FDM	Fluidigm# 3141008B
3	Anti-Mouse CD11c (N418)-142Nd—100 Tests	FDM	Fluidigm# 3142003B
4	Anti-Mouse CD69 (H1.2F3)-143Nd—100 Tests	FDM	Fluidigm# 3143004B
5	Anti-Mouse CD4 (RM4-5)-145Nd—100 Tests	FDM	Fluidigm# 3145002B
6	Anti-Mouse F4/80 (BM8)-146Nd—100 Tests	FDM	Fluidigm#3146008B
7	Anti-Mouse CD103 (2E7)/148Nd-Custom	Custom-X8	Biolegend, #121402
8	Anti-Mouse CD19 (6D5)-149Sm—100 Tests	FDM	Fluidigm#3149002B
9	Anti-Mouse Ly-6C (HK1.4)-150Nd—100 Tests	FDM	Fluidigm #3150010B
10	Anti-Mouse CD25 (3C7)-151Eu—100 Tests	FDM	Fluidigm3151007B
11	Anti-Mouse CD3e (145-2C11)-152Sm—100 Tests	FDM	Fluidigm#3152004B
12	Anti-Mouse CD274/PD-L1-153Eu (10F.9G2)-100 Tests	FDM	Fluidigm#3153016B
13	Anti-Mouse TCRgd (GL3)-154Sm	Custom-X8	Biolegend, #118101
14	Anti-Mouse PD-1 (29F.1A12)-159Tb	FDM	Fluidigm#3159024B
15	Anti-Mouse CD62L (MEL-14)-160Gd—100 Test	FDM	Fluidigm#3160008B
16	Anti-Human/Mouse CD44 (IM7)-162Dy—100 Tests	FDM	Fluidigm#3162030B
17	Anti-Mouse CX3CR1 (SA011F11)-164Dy—100 Tests	FDM	Fluidigm#3164023B
18	Anti-Mouse CD8a (53-6.7)-168Er—100 Tests	FDM	Fluidigm#3168003B
19	Anti-Mouse CD206/MMR (C068C2)-169Tm—100 Tests	FDM	Fluidigm#3169021B
20	Anti-Mouse NK1.1 (PK136)-170Er—100 Tests	FDM	Fluidigm#3170002B
21	Anti-Mouse CD11b (M1/70)-172Yb—100 Tests	FDM	Fluidigm#3172012B
22	Anti-Human/Mouse CD45R/B220 (RA3-6B2)-176Yb—100 Tests	FDM	Fluidigm#3176002B
23	Anti-Mouse I-A/I-E (M5/114.15.2)-209Bi—100 Tests	FDM	Fluidigm#3209006B
24	Anti-Mouse CD127/IL7Ra (A7R34)-175Lu—100 Tests	FDM	Fluidigm#3175006B
